# Stomatal Dimorphism of *Neodiplogaster acaloleptae* (Diplogastromorpha: Diplogastridae)

**DOI:** 10.1371/journal.pone.0155715

**Published:** 2016-05-19

**Authors:** Natsumi Kanzaki

**Affiliations:** Forestry and Forest Products Research Institute, 1 Matsunosato, Tsukuba, Ibaraki, 305–8687, Japan; Laboratoire Arago, FRANCE

## Abstract

Several genera belonging to the nematode family Diplogastridae show characteristic dimorphism in their feeding structures; specifically, they have microbial feeding stenostomatous and predatory eurystomatous morphs. A diplogastrid satellite model species, *Pristionchus pacificus*, and its close relatives have become a model system for studying this phenotypic plasticity, with intensive physiological and structural studies having been undertaken. However, the many other species that are morphologically and phylogenetically divergent from *P*. *pacificus* have not been examined to date. In the present study, the detailed stomatal structure and induction of dimorphism in *Neodiplogaster acaloleptae* were examined. *N*. *acaloleptae* has a fungal feeding stenostomatous morph and a predatory eurystomatous morph. The predatory morph was induced by starvation, high population density, and co-culturing with its potential prey, *Caenorhabditis elegans*. The feeding behavior of the stenostomatous and eurystomatous morphs of *N*. *acaloleptae* was confirmed, demonstrating that 1) the stomatal and pharyngeal movements of the two morphs were basically identical, and 2) the stomatal elements were protracted to cut open the hyphae and/or prey to feed when a *N*. *acaloleptae* flips its dorsal movable tooth dorsally and tilts its subventral stegostomatal cylinder ventrally, forming a pair of scissors to cut the food source. The stoma morphology of *N*. *acaloleptae* with a single movable tooth and a long stoma is markedly different from that of *Pristionchus*, which has two movable teeth and a short stoma. It is, however, similar to that of *Mononchoides*, tentatively a sister to *Neodiplogaster*.

## Introduction

Feeding is one of the most important behaviors of all animals. To ingest nutrients effectively, every animal has evolved feeding structures according to their ecological needs and feeding resources. Therefore, feeding structures can be highly divergent within each animal group. In addition, in some cases, feeding structures differ within a species. For example, the larvae of some lepidopteran and coleopteran insects are grazers that feed on plant material, whereas the adults have a tube-like proboscis to feed on nectar (Lepidoptera) or a brush-like structure to feed on sap flow (Coleoptera) [1, 2]. However, feeding structures do not typically differ within each developmental stage, and relatively few cases of structural polymorphism have been reported [3–5].

The nematode family Diplogastridae is one of those animal groups with polymorphic adult stomatal structures [6–8]. This ancestral characteristic is widespread within the family, and it is often found in genera with complex stomatal morphology to expand the range of their feeding habitats [7]. Therefore, it would be interesting to establish how this differs across the clade, as each difference must be the result of evolutionary tweaking of an existing mechanism. For example, *Pristionchus* spp. in the stenostomatous (narrow stoma) morph usually feed on bacteria, but animals with the eurystomatous (wide stoma) morph occur in starved cultures, and they are effective predators of other nematodes [9, 10].

The stomatal dimorphism of *P*. *pacificus* Sommer, Carta, Kim & Sternberg has been examined as a model of developmental plasticity. Intensive physiological and structural studies on *P*. *pacificus* and its close relatives have revealed that the nervous system, chemical compounds, and chemical receptor genes are involved in the developmental switches that result in functional differences between the two morphs [6, 11–14]. However, the stomatal dimorphism and functional morphology of other diplogastrid nematodes have not been sufficiently examined [14]. Thus, a detailed examination of feeding behavior and ecological characteristics of diplogastrids with stomatal morphology clearly different from *Pristionchus* will help to elucidate further the structural and behavioral aspects of the satellite model system.

The genus *Neodiplogaster* Cobb contains several dimorphic species, which have a microbial feeding stenostomatous morph and a predatory eurystomatous morph [15]. Susoy et al. [7] demonstrated that, in several diplogastrid nematode species, including an undescribed *Neodiplogaster* sp., the eurystomatous morph is induced by co-culture with potential prey [e.g., *Caenorhabditis elegans* (Maupas)]. However, the morphological characteristics of the eurystomatous morphs, with the exception of *N*. *pinicola* Steiner and an undescribed *Neodiplogaster* sp. [7, 15], have not been examined in detail.

*Neodiplogaster acaloleptae* Kanzaki was originally found as a phoretic associate of a longhorn beetle, *Acalolepta luxuriosa* (Bates) (Coleoptera: Cerambycidae) from Shiga Prefecture, Japan [16]. The species was not successfully cultured at the time. It was thus described based on fixed-type material of stenostomatous individuals only, and no molecular profile was obtained. Thereafter, the species was reisolated from the type carrier beetle, *A*. *luxuriosa*, collected from the experimental nursery of the Forestry and Forest Products Research Institute (FFPRI), Ibaraki Japan, and the nematode was successfully cultured on a fungal lawn of *Botrytis cinerea* Pers. During the culturing procedure, the eurystomatous morph appeared in the old (starved) culture and was artificially induced using the methodology of Susoy et al. [7].

The present study describes the eurystomatous morph of *N*. *acaloleptae* and presents a molecular barcode for the species. Environmental factors involved in induction of the eurystomatous morph were examined experimentally, and the feeding behavior was observed using live specimens, focusing on the functional morphology.

## Materials and Methods

### Nematode isolation and culturing

Several adults of *A*. *luxuriosa* that fed on the leaves and twigs of *Aralia elata* (Miq.) were collected at the experimental nursery of the FFPRI, Tsukuba, Ibaraki, Japan (GPS: 36°00’ 22.36”, N, 140°07’ 36.79”, 23 m a.s.l.) on June 24, 2014. The collected insects were dissected and examined for associated nematodes. During the dissection, dauer juveniles of diplogastrid nematodes were found in the tracheal system, genitalia, and ovipositor. The dauer juveniles were transferred to a nematode growth medium (NGM) plate seeded with *Escherichia coli* strain OP50 or malt extract agar (MEA) seeded with *B*. *cinerea*. The successfully propagated cultures were then examined morphologically under a light microscope (Eclipse 80i, Nikon, Tokyo) for species identification. The species associated with the tracheal system propagated on the fungal lawn and was identified as *N*. *acaloleptae*, whereas the other species propagated on bacteria and was identified as a *Diplogasteroides* species that has subsequently been described as *D*. *luxuriosae* Kanzaki & Ide [17]. These two species were subcultured to MEA-*B*. *cinerea* (*N*. *acaloleptae*) or NGM-*E*. *coli* (*D*. *luxuriosae*) medium and maintained as laboratory cultures with the codes NKZ339 and NKZ304, respectively.

None of these nematodes or beetles were endangered or protected species, and all materials were collected on the FFPRI campus. Therefore, no special permits or permissions were required for the present study.

### Molecular profiles and phylogenetic analysis

Nematode lysates were prepared for polymerase chain reaction (PCR) according to Kikuchi et al. [18] and Tanaka et al. [19]. The molecular sequences of the near-full-length small subunit (SSU; 18S) and D2–D3 expansion segments of the large subunit (LSU; 28S) ribosomal RNA genes were sequenced according to Ye et al. [20] and Kanzaki and Futai [21], respectively.

### Induction of the eurystomatous morph

The eurystomatous morph of *N*. *acaloleptae* was induced by co-culturing with *C*. *elegans*, using a modification of the methodology suggested by Susoy et al. [7]. A mixed-stage population of *N*. *acaloleptae* propagated on *B*. *cinerea* in which no eurystomatous morph was found was identified by examination of 200 randomly selected males and females. About 200 mixed-staged individuals, which had been gently washed with sterile water, were inoculated onto a *B*. *cinerea* fungal lawn on PDA in 60-mm-diameter Petri dishes or an *E*. *coli* bacterial lawn on NGM in 60-mm-diameter Petri dishes and incubated at 25°C. In half of these plates, *N*. *acaloleptae* were co-cultured with *C*. *elegans*; that is, *ca*. 100 individuals of mixed-staged *C*. *elegans* were inoculated onto the plate. Then, 1 and 2 weeks after inoculation, nematodes were isolated from the culture plates using the Baerrmann funnel technique [22], and the number, sex ratio, and ratios of the eurystomatous morph in females and males were evaluated.

The sex ratio was calculated as the number of males/females in the first 200 adult individuals, and the eurystomatous ratio was calculated as the percentage of eurystomatous morph individuals among the first 100 females or males. Five plates were examined for each treatment.

All data, average number, sex ratio, and ratios of the eurystomatous morph in females and males were analyzed using StatView 4.0 for Macintosh (SAS Institute, Cary, NC). The normality of the data was examined using a histogram. One-way analysis of variance (ANOVA) followed by Tukey-Kramer’s post hoc test was conducted for comparisons among the treatments. Sex ratio, namely, whether or not there was a bias towards one sex, was examined by the chi-square test.

### Morphological observations and micrographs

Live adults of *N*. *acaloleptae* obtained from the culturing experiments were observed using the methodology of Kanzaki [23]. Several morphological characteristics were drawn, and micrographs were obtained using a drawing tube and digital camera system (DS-Ri1; Nikon, Tokyo) connected to a microscope. The digital micrographs were edited using Photoshop Elements v. 3 computer software (Adobe, San Jose, CA).

Following the morphological observations, the remaining nematodes were heat-killed at 60°C for 1 minute and fixed in TAF (triethanolamine formalin: 7.0% formalin, 2.0% triethanolamine, 91% distilled water) for 48 hours. Nematodes were then dehydrated through a glycerin-ethanol series using a modification of Seinhorst’s method [24], and mounted in glycerin according to the method of Maeseneer and d’Herde [25]. Mounted voucher specimens were used for morphometrics with the aid of a drawing tube.

### Observation of the feeding behavior of the eurystomatous and stenostomatous morphs of *N*. *acaloleptae*

To observe the feeding behavior of the stenostomatous morph, a small number of fungal hyphae and several stenostomatous adult females were mounted alive on an agar pad and observed under a light microscope. Predation by the eurystomatous morph was observed using a modification of the methodology suggested by Fürst von Lieven [26]; that is, dauer juveniles of *C*. *elegans* and eurystomatous females of *N*. *acaloleptae* were collected on a 2.0% agarose layer of 3-mm thickness, a cover slip was placed over the nematodes, the agar was cut along the edge of the cover slip, and the cut piece of agar and cover slip were transferred to a glass slide for microscopic observation. Videos of the feeding behavior were captured using a digital microscope camera system, MC170 HD (Leica Microsystems, Wetzlar, Germany).

## Results

### Induction of the eurystomatous morph

The average number, sex ratio, and eurystomatous ratios of females and males of *N*. *acaloleptae* are summarized in Table 1. The sex ratio was slightly skewed towards females; that is, the male/female ratio was usually <0.9, irrespective of the culture conditions (P < 0.05). The average number of nematodes was markedly higher on *B*. *cinerea* plates than on *E*. *coli* plates, but they were similar in corresponding monoculture and co-culture with *C*. *elegans*. However, the percentages of eurystomatous females and males were higher in co-cultures compared with in the corresponding monocultures (Table 1).

**Table 1:** Average number, sex ratio, and proportions of the eurystomatous morph in females and males of *Neodiplogaster acaloleptae* under different culture conditions.

Culture period	Culture condition			Number of nematodes	Sex ratio	% eurystomatous females	% eurystomatous males
	fungi	bacteria	prey				
**1 week**	+	-	-	15,781^a^ ± 4,053	0.81^a^ ± 0.06	0.8^d^ ± 0.8	0.2^b^ ± 0.4
	+	-	+	12,640^a^ ± 3,981	0.85^a^ ± 0.30	15.6^c^ ± 11.8	8.6^b^ ± 2.9
	-	+	-	252^d^ ± 102	0.69^a^ ± 0.17	13.5^c^ ± 18.6	1.3^b^ ± 2.8
	-	+	+	322^d^ ± 121	0.71^a^ ± 0.12	96.3^a^ ± 2.7	48.6^a^ ± 12.0
**2 weeks**	+	-	-	8,760^b^ ± 1,677	0.57^a^ ± 0.16	5.6^d^ ± 4.1	2.4^b^ ± 1.5
	+	-	+	7,110^b^ ± 1,380	0.43^a^ ± 0.09	68.0^b^ ± 2.3	27.2^ab^ ± 17.0
	-	+	-	1,341^c^ ± 356	0.88^a^ ± 0.08	2.3^d^ ± 2.2	0^b^ ± 0.0
	-	+	+	545^d^ ± 317	0.80^a^ ± 0.15	62.1^b^ ± 23.8	51.4^a^ ± 29.6

All values are in the form: mean ± standard deviation (sd). Means followed by the same letter in a column are not significantly different (P < 0.05).

### Morphology of the eurystomatous morph

**Fig 1:**
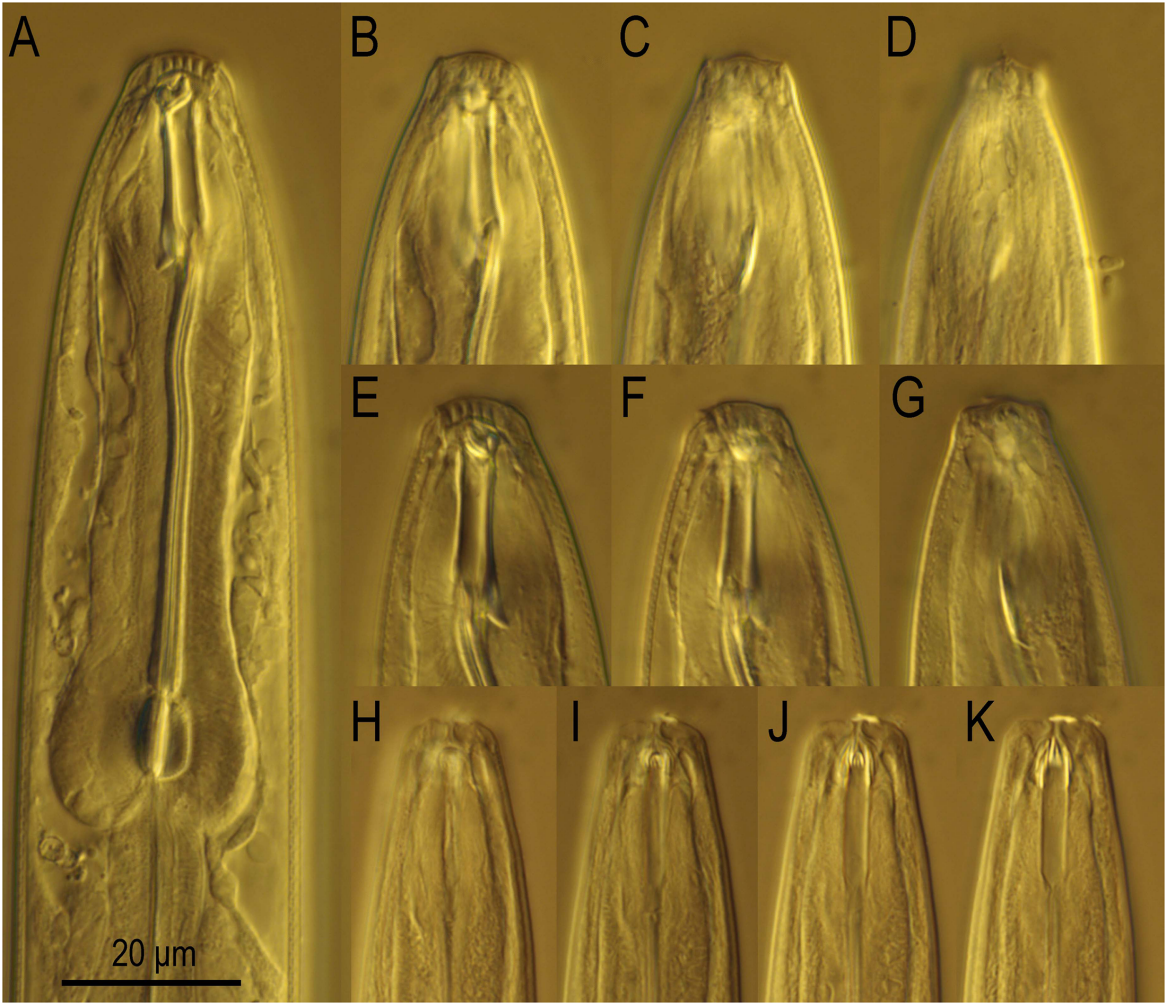
The anterior part of eurystomatous females of *Neodiplogaster acaloleptae*. DIC (differential interference contrast) microphotographs. A: Right lateral view of the anterior end to the anterior pharynx. B–D: Right lateral view of the stomatal region in different focal planes. E–G: Left lateral view of the stomatal region in different focal planes. H–K: Ventral view of the stomatal region in different focal planes. A and B–D are from the same individual.

**Fig 2:**
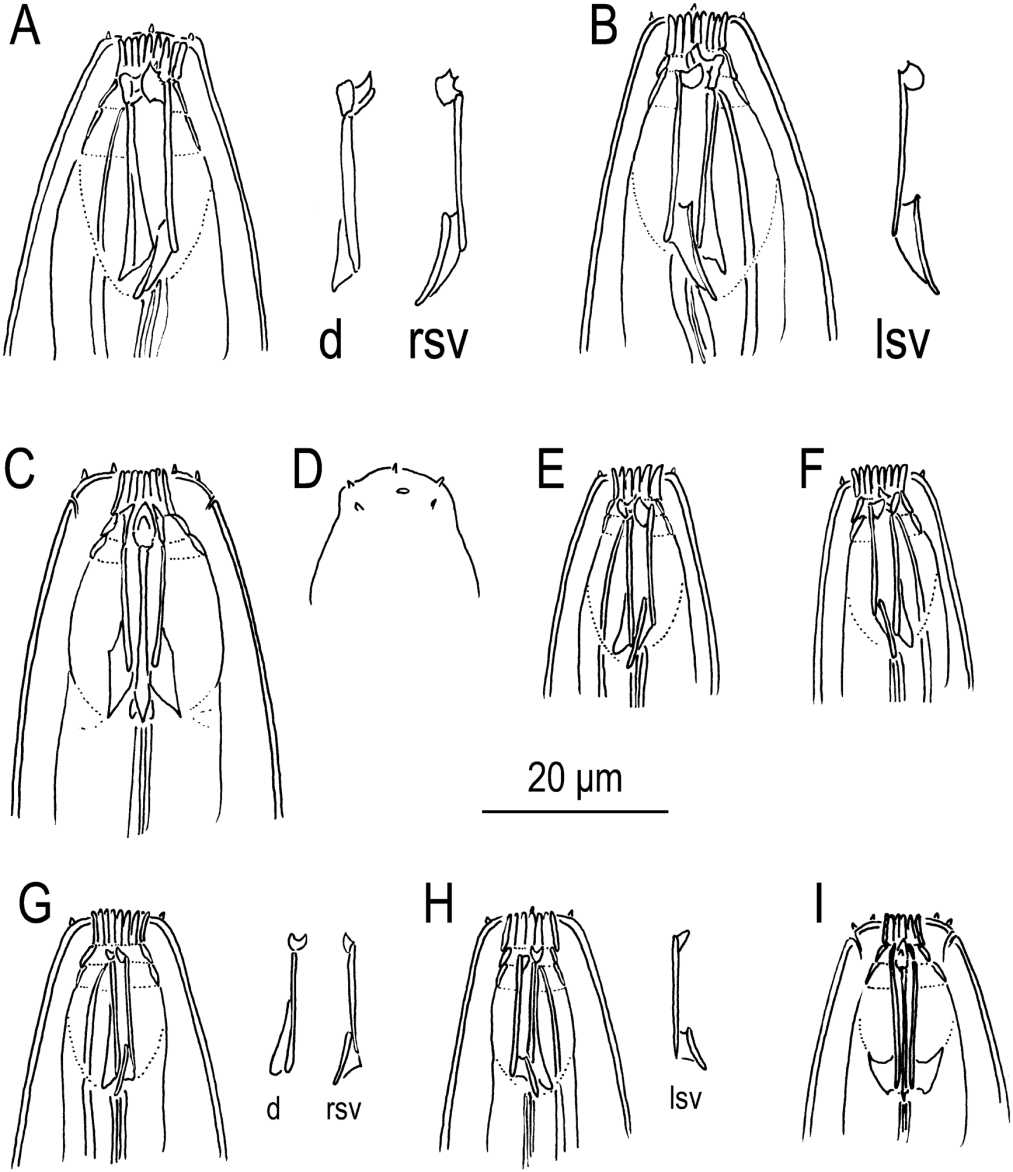
The stomatal region of *Neodiplogaster acaloleptae*. **Drawings.** A-F: Eurystomatous morph. A: Right lateral view of a female (d = dorsal meta-telostegostomatal element, rsv = right subventral meta-telostegostomatal element). B: Left lateral view of a female (lsv = left subventral meta-telostegostomatal element). C: Ventral view of a female. D: Anterior body surface of a male showing labial sensilla, cephalic papillae, and amphid. E: Right lateral view of a male. F: Left lateral view of a male. G-I: Stenostomatous morph. G: Right lateral view of a female. H: Left lateral view of a female. I: Ventral view of a female.

The body size of the eurystomatous morph was slightly smaller than that of the stenostomatous morph; however, its general morphology, with the exception of the stomatal region, was not clearly different from that of the stenostomatous morph. Therefore, the stomatal morphology of the eurystomatous morph is described below (Figs 1 and 2). Morphometric data for the eurystomatous and stenostomatous males and females from this study and from the original description (stenostomatous morph) are provided in S1 Table.

#### The stomatal structure of the eurystomatous morph

From the anterior, the stoma was separated into three sections: the cheilostom, gymnostom, and stegostom. The stegostom was separated into three subsections: the pro-meso stegostom, metastegostom, and telostegostom. The cheilostom was short and tube-like, with a width about twice its depth, likely separated into 18 narrow plates (= rugae). The gymnostom was short, ring-like, anteriorly narrowing, and slightly shorter in length than the cheilostom. The interior part of the gymnostom internally overlapped with the posterior part of the cheilostom. The stegostom was deep, appearing as a cuticular cylinder. The pro-mesostegostom was difficult to observe, namely, it was thin, forming a dome-shaped cuticular tube, and a little shorter in length than the cheilostom. The metastegostom formed a square-shaped movable dorsal tooth, a square-shaped right subventral tooth with several spinous processes, and a rounded left subventral ridge; both teeth and a ridge were connected to the stegostomatal cylinder (= telostegostom). The dorsal telostegostom had a triangular dorsal apodeme. The right and left subventral telostegostoms bore wing- or parallelogram-shaped subdorsally oriented apodemes. Connections of the myofilaments to the apodemes and tooth were not clearly observed. The dorsal pharyngeal gland extended to the dorsal tooth, and the gland duct penetrated the dorsal tooth to the buccal cavity. Although the basic composition of stomatal morphology was identical in males and females, the stomatal diameter was smaller in males, and the eurystomatous and stenostomatous morphs in males were sometimes difficult to distinguish.

#### Some additional morphological characteristics of *N*. *acaloleptae* found during microscopic observation of the live materials

Small triangular right and left subventral projections homologous to the eurystomatous right subventral tooth and left subventral ridge were found at the metastegostom of the stenostomatous morph. The hemizonid was faintly visible slightly anterior to the excretory pore. The presence of a deirid at the level of the cardia was confirmed. The postdeirid was found at the level of the middle of the *vas deferens* of males and the posterior end of the posterior ovary of females. It was confirmed that the *vas deferens* structure of males possessed a glandular part at the junction between the testis and the *vas deferens*; the anterior part was composed of large and rounded cells, and the posterior part had a thin wall. The male phasmid was located near the v5–7 genital papillae. These characteristics are shown in S1 Fig.

### Voucher materials

The mounted materials examined for their morphometric values were deposited in the laboratory collection of the FFPRI with the slide numbers *Neodiplogaster acaloleptae* Mv01–Mv40 and Fv01–Fv40.

In addition to the mounted voucher materials, the TAF-fixed materials and cultured material of *N*. *acaloleptae* (NKZ339) are available at the Forest Pathology Laboratory (N. Kanzaki) and can be provided to other researchers upon request.

### Molecular profiles

Molecular sequences for *N*. *acaloleptae* were deposited in the GenBank database under the accession numbers LC107877 (near-full-length of SSU) and LC107878 (D2-D3 expansion segments of LSU).

### Feeding behavior of *N*. *acaloleptae*

Videos of the stenostomatous individuals on fungi and dead nematode bodies are available as supplemental films (S1 and S2 videos), as is a video of the eurystomatous individuals feeding on *C*. *elegans* (S3 video).

**Fig 3:**
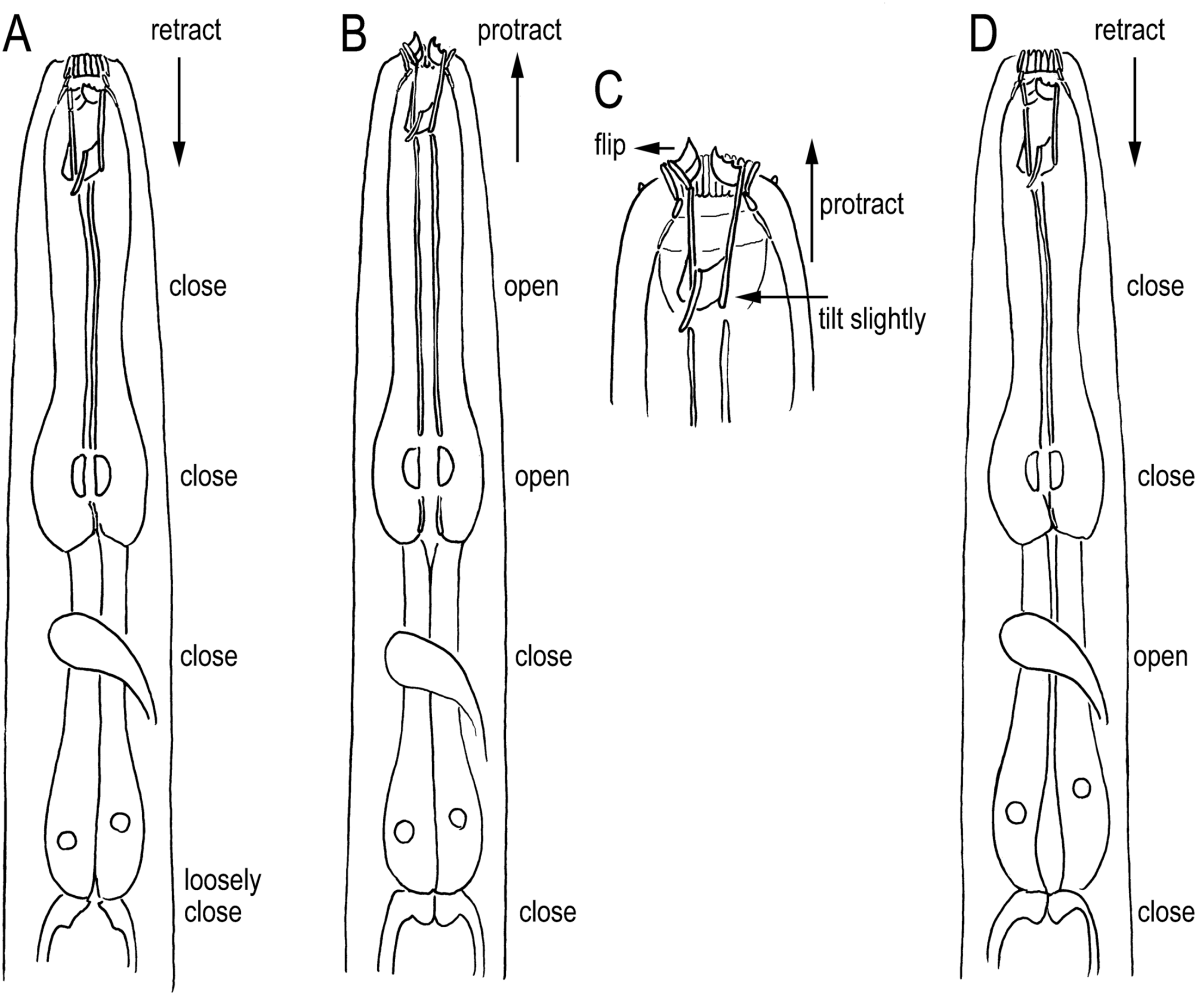
Schematic drawing of the stomatal and pharyngeal movement of *Neodiplogaster acaloleptae* from a right lateral view. A: Anterior part at rest when the stoma is retracted. Anterior and posterior parts of the pharynxe are closed, and the cardia is loosely closed (or open). B: Protraction of stomatal elements (cutting and sucking of the food source). When the stoma is protracted by extension of the procorpus, the anterior pharynx is opened and the posterior pharynx and cardia are closed. C: Close-up of the stomatal region of B, where the cheilostomatal ring (rugae) is widened slightly, the dorsal tooth is flipped dorsally, and subventral elements are tilted slightly ventrally. D: Ingestion of food when the stoma is retracted. The anterior pharynx is closed and the posterior pharynx is open to transfer the food to the cardia.

The feeding of both morphs comprised three movements: 1) protraction of the stomatal elements (cheilostom, gymnostom, and stegostom) during which the dorsal tooth was flipped (or pushed) out to widen the stomatal opening, the subventral metastegostomatal and telostegostomatal elements were tilted slightly ventrally, the anterior pharynx (procorpus + metacorpus) was opened simultaneously, and the posterior pharynx (isthmus + basal bulb) and cardia were closed; 2) retraction of the stomatal elements, flipping back of the dorsal tooth and closing of the anterior pharynx, and then opening of the posterior pharynx; and 3) closure of the posterior pharynx and opening of the cardia to transfer the food to the intestine (Fig 3). If the contents of a prey nematode leaked out through a cut in the cuticle made by the predator, *N*. *acaloleptae* kept the stoma open (Fig 3B and 3C) and moved only the pharynx to suck up the food. The movements of stoma and pharynx were more active when *N*. *acaloleptae* individuals fed on dead or alive nematodes than when they fed on fungal hyphae.

The movements of the stoma and anterior pharynx appeared to be well coordinated, that is, they moved simultaneously. Additionally, the movements of the posterior pharynx and cardia seemed coordinated with each other. However, the coordination of the anterior part (stoma + anterior pharynx) and posterior part (posterior pharynx + cardia) was a little less stringent; that is, the posterior part sometimes did not open immediately after the anterior part closed.

## Discussion

### Food sources and induction of the eurystomatous morph

Previous studies have considered the genus *Neodiplogaster* to be an omnivorous group [15, 27]. The preferred food can vary among species. For example, *N*. *acaloleptae* and *N*. *crenatae* Kanzaki, Masuya, & Kubono were successfully cultured on a fungal lawn but not on a bacterial one [13], whereas an undescribed *Neodiplogaster* sp. has long been cultured on bacteria in the laboratory [7]. Similarly, some *Mononchoides* species, most of which are presumed to be predators [27], could be cultured on bacterial lawns [7, 28].

The eurystomatous morph is known to be induced by starvation, high population density, and/or co-culturing with its potential prey [7]. In *N*. *acaloleptae*, the proportion of the eurystomatous morph usually increased after 1 week of culture, and it increased further after 2 weeks of culture. Additionally, co-culture with a prey nematode increased the proportion of eurystomatous worms compared with the corresponding monoculture treatment. The nematode did not propagate well in monocultures on bacterial lawns, and the effect of population density can likely be neglected; that is, the high ratio of the eurystomatous morph was likely induced purely by starvation (Table 1).

**Fig 4:**
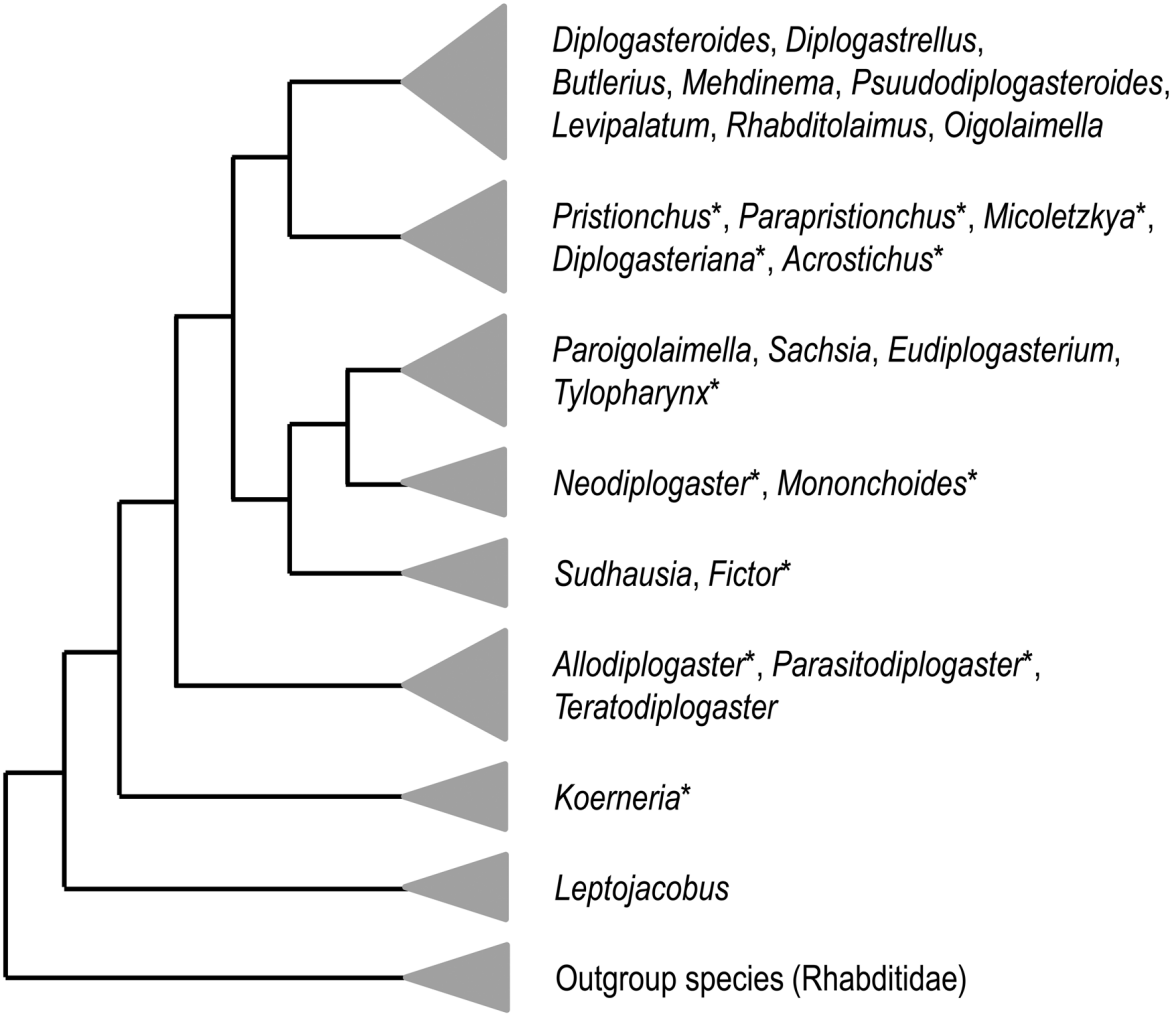
Phylogenetic relationships among diplogastrid genera. The phylogenetic tree provided by Susoy et al. [7] was simplified to show the relationships among groups of specified genera. The size of a triangle indicates the number of genera included in each clade. An asterisk indicates a genus with stomatal dimorphism.

Thus, three factors—starvation, high population density, and co-culturing—seem to induce the eurystomatous morph in *N*. *acaloleptae*. Susoy et al. [7] demonstrated that co-culturing strongly induces the eurystomatous morph in *Allodiplogaster*, *Fictor*, *Micoletzkya*, *Mononchoides*, and *Neodiplogaster*, but not in *Diplogasteriana*, *Koerneria*, *Parapristionchus*, or *Pristionchus*. Interestingly, sensitivity to co-culturing is not clearly correlated with the phylogenetic relationship (Fig 4). In the present and previous studies [7], the eurystomatous morph of these nematodes was induced by the presence of potential prey, *C*. *elegans* [29]. Although the precise chemical cues have not been identified in most diplogastrids, except for *P*. *pacificus* [12], the conditions for the induction of stomatal polymorphism seem to vary among these genera. Detailed examination of the natural prey and chemical analysis based on the chemical profiles, for example, the pheromone profile [30], of potential prey nematodes should be performed to obtain a deeper understanding of the ecological aspects of the eurystomatous morph.

The proportion of eurystomatous morphs was apparently higher in females than in males, and a similar phenomenon has been observed in other diplogastrid nematodes: for example, the eurystomatous morph has been observed only in females in *Allodiplogaster* spp. [31] and *Acrostichus* spp. [32, 33]. These genera are not phylogenetically close to *Neodiplogaster*; thus, the more frequent occurrence of the eurystomatous morph in females may be a general tendency in the family.

### Functional morphology of stoma and pharynx

The functional morphology of feeding structures has been examined in several diplogastrid species. Fürst von Lieven [26] examined the movement of stomatal elements of *Tylopharynx foetida* (Bütschli) and *Mononchoides* spp., and Bumbarger [34], Serobyan et al. [9, 10], Ragsdale et al. [6] and Wilecki et al. [14] examined the detailed feeding pattern of both the stenostomatous and the eurystomatous morphs of *Pristionchus pacificus* and some other diplogastrid nematodes. The feeding behavior of the eurystomatous morph of *P*. *pacificus* [34] can be viewed online (https://www.youtube.com/watch?v=vT8bms2RHe8). The feeding pattern seems to differ among these species; that is, the functional morphologies of the feeding structure of the species (genera) with two movable teeth seem to be similar, but those having a single movable tooth (*Tylopharynx*, *Mononchoides*, and *Neodiplogaster*) seem to differ from each other, and from the species with two movable teeth.

The eurystomatous morph of *P*. *pacificus* (and other *Pristionchus* spp.) has wide stoma and large dorsal and right subventral movable teeth. In a video and observations made available previously [14, 34], the movements of the stoma and pharynx of eurystomatous *Pristionchus* and some other species (*Parapristionchus*, *Allodiplogaster*, and *Micoletzkya*) were as follows. First, the nematode catches prey by sucking its surface into the stoma. This occurs by contacting the prey, opening the anterior pharynx to generate negative pressure, and then flipping the two teeth like a pair of scissors to cut open the cuticle. Thereafter, the body contents of the prey are sucked out by an opening/closing movement of the pharynx, during which the movement of teeth seems to be independent of the movement of the anterior pharynx; that is, the teeth move occasionally for mastication, but they open when the pharynx opens. During the sequence of movements, eurystomatous *Pristionchus* does not show obvious protraction of stomatal elements, probably because its wide stoma is adapted to sucking out the contents of the prey. Interestingly, these four genera show clear differences in feeding behavior between the stenostomatous and eurystomatous morphs [9, 10, 14]. In these genera, as typified by *P*. *pacificus*, the stenostomatous morph has only one movable tooth (small dorsal tooth), and the tooth is located at the bottom of the stoma. Therefore, the tooth basically moves like a flap to help take in bacteria, while the other stomatal elements do not appear to move [9, 10, 14]. This clear functional difference between the two stomatal morphs was not observed in *Neodiplogaster*, which basically has very similar but slightly differently scaled stomatal composition in the two morphs.

**Fig 5:**
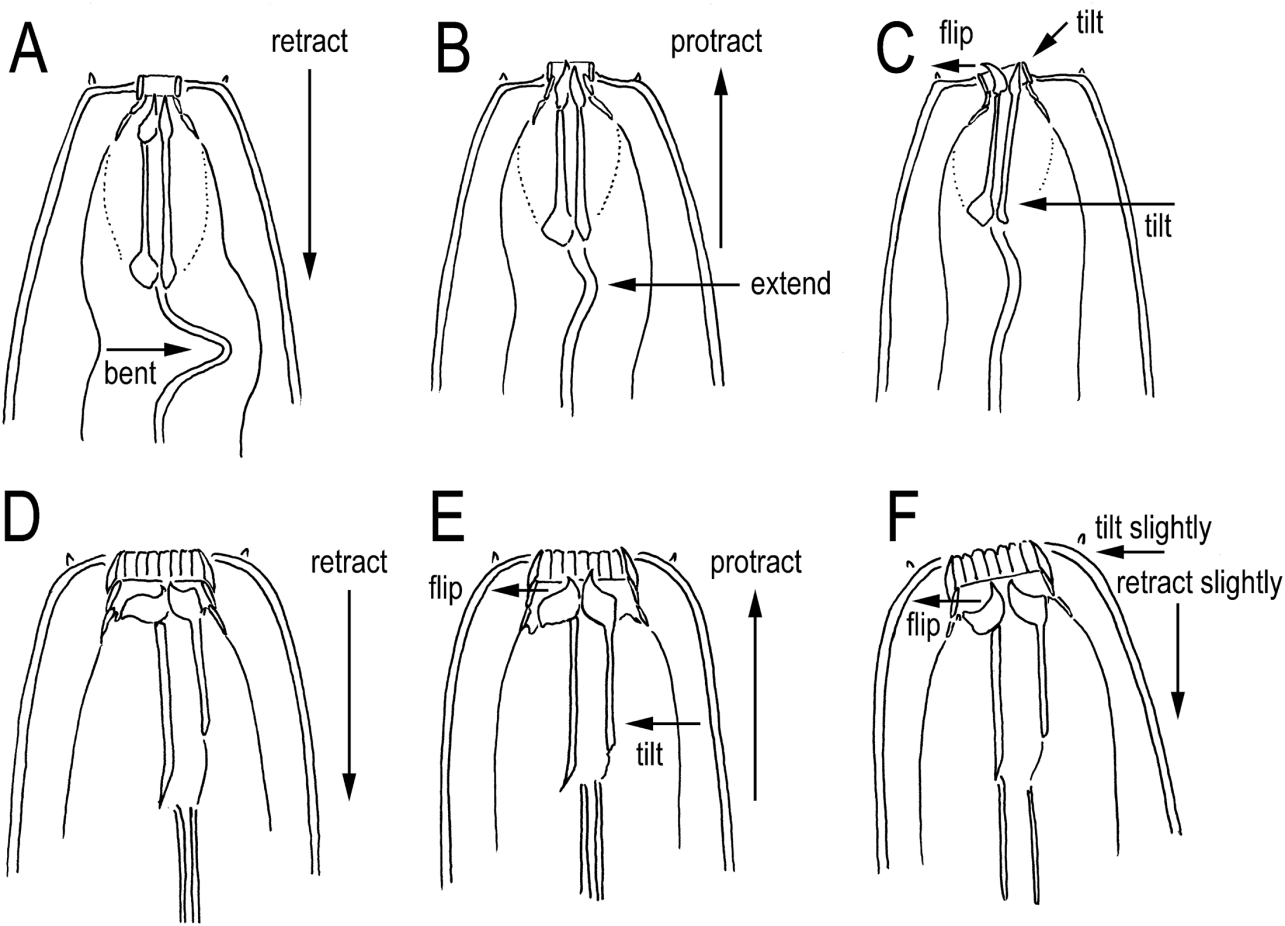
Schematic drawing of the stomatal movement of *Tylopharynx* and *Mononchoides* from a right lateral view. Figures were redrawn based on those provided by Fürst von Lieven [26]. A–C: *Tylopharynx*. A: At rest when the stoma is retracted. B: Protraction of stomatal elements by extending the pharyngeal tube (spearing the fungal hypha). C: The cheilostomatal ring tilts slightly dorsally, the dorsal tooth is flipped dorsally, and the subventral elements are tilted slightly ventrally (cutting open the hypha to suck up the hyphal contents). D–F: *Mononchoides*; D: At rest when the stoma is retracted. E: Protraction of stegostomatal elements where the dorsal tooth is slightly flipped dorsally, and subventral elements are tilted slightly ventrally (biting the prey). F: Ingestion of food retracting the stegostomatal elements where the head is tilted slightly dorsally, and the dorsal tooth is strongly flipped dorsally to open the stoma.

On the other hand, *Mononchoides* spp., which also have a wide stoma, show slight protraction of the gymnostom and stegostom [26] (Fig 5). Because the right subventral tooth of *Mononchoides* spp. is connected to the subventral stegostomatous cylinder, the cylinder must be tilted to move the tooth [26]. Therefore, *Mononchoides* spp. must suck the prey into their stoma and move the stomatal elements slightly forward, tilting the subventral cylinder and flipping the dorsal tooth to cut open the prey.

The feeding of *Tylopharynx* de Man, which has a narrow stoma, clearly differs from that of the above two genera (Fig 5). Because of its narrow stoma, inflexible cheilostomatal ring, and procorpus forming a narrow procorpal tube, *Tylopharynx* cannot effectively suck its food (hyphae) into the stoma, and the teeth are too small to reach the stomatal opening even when they are flipped. Thus, these nematodes protract the gymnostomatal and stegostomatal elements to outside of the stoma. The feeding of *Tylopharynx* comprises three movements: 1) protraction of the gymnostom to push it onto the hyphae; 2) protraction of the stegostomatal elements extending the procorpal tube to insert the teeth into the hyphae; and 3) tilting of the gymnostom dorsally, tilting of the stegostomatal cylinder ventrally, and flipping of the dorsal tooth to cut open the hyphae to suck out the contents [26]. Thus, the typical movements in *Tylopharynx*, namely, the extension of the procorpal tube and tilting of the cheilostom, were not observed in *Neodiplogaster* and *Mononchoides* [26].

The feeding behavior of *N*. *acaloleptae* seems somewhat intermediate between those of *Mononchoides* and *Tylopharynx*, but it is more similar to that of *Mononchoides* spp. Comparing *Neodiplogaster* with *Monochoides*, the protraction of the stoma is much clearer in *Neodiplogaster*, and the dorsal tilt of the anterior end seen in *Monochoides* (Fig 5F) is somewhat unclear in *Neodiplogaster*. Specifically, *Neodiplogaster* expands the cheilostom and the teeth protrude from the stoma, whereas this does not occur in *Monochoides* [26]. This extensive protraction may be needed to access the prey since the stoma of *Neodiplogaster* is considerably narrower than that of *Mononchoides*, even in the eurystomatous morph. Feeding in *Neodiplogaster* and *Monochoides* differs from feeding in *Tylopharynx* in that protraction and ventral tilt of the stoma happens almost at the same time in these species (Fig 5E). In contrast, in *Tylopharynx*, protraction is followed by the tilt in a second step (Fig 5B and 5C). These differences could be linked to the difference in the width and flexibility of the cheilostom, which is wider and separated into rugae in *Mononchoides* and *Neodiplogaster* but is a narrow inflexible ring in *Tylopharynx* (Figs 3C, 5B, 5C, 5E and 5F).

Susoy et al. [7] suggested that *Mononchoides* and *Neodiplogaster* are phylogenetically close and that *Tylopharynx* and several other diplogastrids form a sister clade of the *Mononchoides*/*Neodiplogaster* clade (Fig 4); that is, *Neodiplogaster* shares a narrow stylet-like stoma with *Tylopharynx*, but is phylogenetically closer to *Mononchoides*. The similarity in the feeding structure and behavior of *Mononchoides* and *Neodiplogaster* seems in accordance with the phylogenetic relationship of these genera. At present, the origin of the narrow stoma shared by the above two genera is unclear, namely, whether this occurred independently in the two genera or was an apomorphic characteristic of their common ancestor. More detailed molecular phylogenetic and morphological characteristic studies may enhance our understanding of the origin and evolutionary history of the stomatal structure of *N*. *acaloleptae*.

## Supporting Information

S1 Fig Newly confirmed morphological characteristics of *Neodiplogaster acaloleptae*. A: Left lateral view of the anterior part of a female showing the hemizonid and deirid. B: Postdeirid position of the female. C: The gonad and postdeirid of the male. D: Close-up of the male postdeirid. E: Ventral view of the male tail tip showing ad, pd, and v5–7 papillae and phasmid position.
(TIF)

S1 Table Morphometric values of *Neodiplogaster acaloleptae*. All measurements are in μm and in the form: mean ± standard deviation (sd; range).
(DOCX)

S1 Video Stenostomatous morph of *Neodiplogaster acaloleptae* feeding on fungal hyphae. The stoma and pharynx coordinate with each other to cut open the hyphae and take in the hyphal contents.
(MP4)

S2 Video Stenostomatous morph of *N*. *acaloleptae* feeding on the body of a dead nematode. Stoma and pharynx movements are basically the same as in fungal feeding; however, sometimes the stoma is kept open, and the pharynx is moved to take in the contents of a dead nematode.
(MP4)

S3 Video A eurystomatous morph of *N*. *acaloleptae* predating on a *Caenorhabditis elegans* juvenile. The stoma is kept open and the pharynx keeps moving while sucking in the leakage (0–10 s). The coordination of stoma and pharynx is clearly observed from 40–50 s.
(MP4)

## References

[1] Krenn HW (2010) Feeding mechanisms of adult Lepidoptera: structure, function, and evolution of the mouthparts. Ann Rev Entomol 55: 307–327. 19961330 10.1146/annurev-ento-112408-085338 PMC4040413

[2] Tanahashi M, Matsushita N, Togashi K (2009) Are stag beetles fungivorous? J Insect Physiol 55: 983–988. 10.1016/j.jinsphys.2009.07.002 19607834

[3] Pfennig DW, Murphy PJ (2002) How fluctuating competition and phenotypic plasticity mediate species divergence. Evolution 56: 1217–1228. 12144021 10.1111/j.0014-3820.2002.tb01433.x

[4] Nijhout HF (2003) Development and evolution of adaptive polymorphisms. Evol Dev 5: 9–18. 12492404 10.1046/j.1525-142x.2003.03003.x

[5] Pfennig DW, Wund MA, Snell-Rood EC, Cruichshank T, Schlichting CD, Moczek AP (2012) Phenotypic plasticity’s impacts on diversification and speciation. Trends Ecol Evol 25: 459–466. 10.1016/j.tree.2010.05.006 20557976

[6] Ragsdale EJ, Müller MR, Rödelsperger C, Sommer RJ (2013) A developmental switch coupled to the evolution of plasticity acts through a sulfatase. Cell 155: 922–933. 10.1016/j.cell.2013.09.054 24209628

[7] Susoy V, Ragsdale EJ, Kanzaki N, Sommer RJ (2015) Rapid diversification associated with a macroevolutionary pulse of developmental plasticity. eLife 4: e05463. 10.7554/eLife.05463 PMC4357287 25650739

[8] Susoy V, Herrmann M, Kanzaki N, Kruger M, Nguyen CN, Rödelsperger C, et al (2016) Large-scale diversification without genetic isolation in nematode symbionts of figs. Sci Adv 2: e1501031 10.1126/sciadv.1501031 26824073 PMC4730855

[9] Serobyan V, Ragsdale EJ, Sommer RJ (2014) Adaptive value of a predatory mouthform in a dimorphic nematode. Proc R Soc B 281: 20141334 10.1098/rspb.2014.1334 25080344 PMC4132687

[10] Serobyan V, Ragsdale EJ, Müller MR, Sommer RJ (2013) Feeding plasticity in the nematode *Pristionchus pacificus* is influenced by sex and social context and is linked to developmental speed. Evol Dev 15: 161–170. 10.1111/ede.12030 23607300

[11] Bento G, Ogawa A, Sommer R J (2010) Co-option of the endocrine signalling module Dafachronic Acid-DAF-12 in nematode evolution. Nature 466: 494–497. 10.1038/nature09164 20592728

[12] Bose N, Ogawa A, von Reuss SH, Yim JJ, Ragsdale E J, Sommer RJ, et al (2012) Complex small molecular architectures regulate phenotypic plasticity in a nematode. Angew Chem 51: 12438–12443. 23161728 10.1002/anie.201206797 PMC3733369

[13] Bumbarger DJ, Riebesell M, Rödelsperger C, Sommer RJ (2013) System-wide rewiring underlies behavioral differences in predatory and bacterial-feeding nematodes. Cell 152: 109–119. 10.1016/j.cell.2012.12.013 23332749

[14] Wilecki M, Lightfoot J, Susoy V, Sommer RJ (2015) Predatory feeding behavior in *Pristionchus* nematodes is dependent on a phenotypic plasticity and induced by serotonin. J Exp Biol 218: 1306–1313. 10.1242/jeb.118620 25767144

[15] Hechler HC (1971) Redescription of *Neodiplogaster tropica* Cobb and *N*. *pinicola* Steiner (Nematoda: Diplogasteridae). J Nematol 3: 341–348. 19322389 PMC2619905

[16] Kanzaki N, Masuya H, Kubono T (2008) Description of *Neodiplogaster crenatae* sp. n. and *N*. *acaloleptae* sp. n. (Nematoda: Diplogastridae) from Japan. Nematology 10: 545–560.

[17] Kanzaki N, Ide T (2016) *Diplogasteroides luxuriosae* n. sp. associated with *Acalolepta luxuriosa* (Cerambycidae) from Japan. Nematology 18: 221–233.

[18] Kikuchi T, Aikawa T, Oeda Y, Karim N, Kanzaki N (2009) A rapid and precise diagnostic method for detecting the pinewood nematode *Bursaphelenchus xylophilus* by loop-mediated isothermal amplification (LAMP). Phytopathology 99: 1365–1369. 10.1094/PHYTO-99-12-1365 19900002

[19] Tanaka R, Kikuchi T, Aikawa T, Kanzaki N (2012) Simple and quick methods for nematode DNA preparation. Appl Entomol Zool 47: 291–294.

[20] Ye W, Giblin-Davis RM, Braasch H, Morris K, Thomas WK (2007) Phylogenetic relationships among *Bursaphelenchus* species (Nematoda: Parasitaphelenchidae) inferred from nuclear ribosomal and mitochondrial DNA sequence data. Mol Phylogenet Evol 43: 1185–1197. 17433722 10.1016/j.ympev.2007.02.006

[21] Kanzaki N, Futai K (2002) A PCR primer set for determination of phylogenetic relationships of *Bursaphelenchus* species within *xylophilus* group. Nematology 4: 35–41.

[22] Hooper DJ (1986) Extraction of nematodes from plant material In: Southey JF ed. Laboratory methods for work with plant and soil nematodes. London: Her Majesty’s Stationery Office pp 51–58.

[23] Kanzaki N (2013) Simple methods for morphological observation of nematodes. Nematol Res 43: 9–13.

[24] Minagawa N, Mizukubo T (1994) A simplified procedure of transferring nematodes to glycerol for permanent mounts. Jpn J Nematol 24: 75.

[25] Hooper DJ (1986) Handling, fixing, staining and mounting nematodes In: Southey JF ed. Laboratory methods for work with plant and soil nematodes. London: Her Majesty’s Stationery Office pp 59–80.

[26] Fürst von Lieven A (2002) Functional morphology, origin and phylogenetic implications of the feeding mechanism of *Tylopharynx foetida* (Nematoda: Diplogastrina). Russ J Nematol 10: 11–23.

[27] Yeates GW, Bongers T, De Goede RGM, Freckman DW, Georgieva SS (1993) Feeding habits in nematode families and genera—an outline for soil ecologists. J Nematol 25: 315–331. 19279775 PMC2619405

[28] Steel H, Moens T, Scholaert A, Boshoff MC, Houthoofd W, Bert W (2011) *Mononchoides composticola* n. sp. (Nematoda: Diplogastridae) associated with composting processes: morphological, molecular and autecological characterisation. Nematology 13: 347–363.

[29] Kiontke KC, Félix M-A, Ailion M, Rockman MV, Braendle C, Pénigault J-B, et al (2011) A phylogeny and molecular barcodes for *Caenorhabditis*, with numerous new species from rotting fruits. BMC Evol Biol 11: 339 10.1186/1471-2148-11-339 22103856 PMC3277298

[30] Braendle C (2012) Pheromones: evolving language of chemical communication in nematodes. Curr Biol 22: R294–R296. 10.1016/j.cub.2012.03.035 22575463

[31] Kanzaki N, Giblin-Davis RM, Ragsdale EJ (2015) *Allodiplogaster josephi* n. sp. and *A*. *seani* n. sp. (Nematoda: Diplogastridae), associates of soil-dwelling bees in the eastern USA. Nematology 17: 831–863.

[32] Giblin RM, Kaya HK (1984) *Aduncospiculum halicti* n. gen., n. sp. (Diplogasterida: Diplogasteroididae), and associate of bees in the genus *Halictus* (Hymenoptera: Halictidae). Rev Nématol 7: 189–197.

[33] Kanzaki N, Giblin-Davis RM, Zeng Y, Ye W, Center BJ, Thomas WK (2009) *Acrostichus puri* n. sp. (Nematoda: Diplogastridae), a phoretic associate of *Augochlora pura mosieri* Cockerell (Hymenoptera: Halictidae). Nematology 12: 49–64.

[34] Bumbarger DJ (2013) P. pacificus eats C. elegans! https://www.youtube.com/watch?v=vT8bms2RHe8 (accessed July 20, 2015)

