# Supplementary figures and images for: Stomatal Dimorphism of Neodiplogaster acaloleptae (Diplogastromorpha: Diplogastridae)
Source: PLoS One. 2016 May 19;11(5):e0155715. doi: 10.1371/journal.pone.0155715 (PMC4873264; doi:10.1371/journal.pone.0155715)

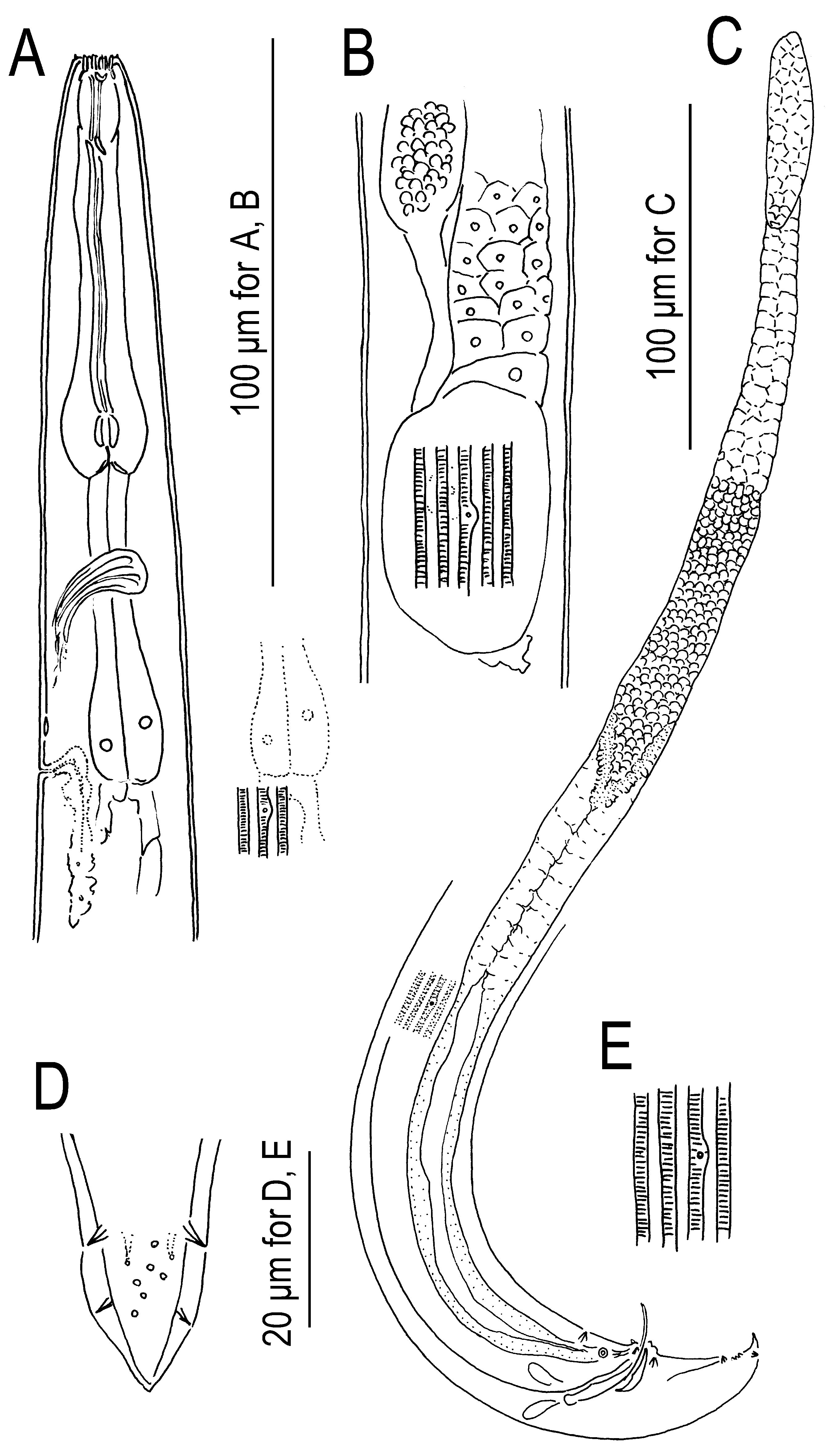

Supplement: S1 Fig — A: Left lateral view of the anterior part of a female showing the hemizonid and deirid. B: Postdeirid position of the female. C: The gonad and postdeirid of the male. D: Close-up of the male postdeirid. E: Ventral view of the male tail tip showing ad, pd, and v5–7 papillae and phasmid position. (TIF) [file pone.0155715.s001.tif]
