# Supplementary material for: Stomatal Dimorphism of Neodiplogaster acaloleptae (Diplogastromorpha: Diplogastridae)
Source: PLoS One. 2016 May 19;11(5):e0155715. doi: 10.1371/journal.pone.0155715 (PMC4873264; doi:10.1371/journal.pone.0155715)
Supplement: S1 Table — All measurements are in μm and in the form: mean ± standard deviation (sd; range). (DOCX) [file pone.0155715.s002.docx]

**S1 Table.** Morphometric values of *Neodiplogaster acalolepatae*.

|  | Eurystomatous form | | Stenostomatous form | | Original description  (paratype stenostomatous form) | |
| --- | --- | --- | --- | --- | --- | --- |
|  | Female | Male | Female | Male | Female | Male |
| n | 20 | 20 | 20 | 20 | 20 | 21 |
| L | 737 ± 53  (645–878) | 716 ± 39  (621–793) | 960 ± 87  (820–1120) | 746 ± 40  (683–800) | 1284 ± 148  (1013–1483) | 802 ± 61  (715–913) |
| a | 21.0 ± 2.5  (17.6–26.0) | 25.2 ± 3.1  (21.1–33.1) | 26.0 ± 2.1  (21.9–30.1) | 26.2 ± 1.8  (23.4–29.3) | 34.0 ± 2.0  (30.2–37.6) | 28.4 ± 2.5  (25.2–35.7) |
| b | 5.1 ± 0.3  (4.6–5.7) | 5.0 ± 0.2  (4.6–5.6) | 6.6 ± 0.4  (5.9–7.5) | 5.5 ± 0.2  (5.0–5.9) | 8.6 ± 0.8  (7.0–9.6) | 6.1 ± 0.4  (5.3–7.0) |
| c | 5.3 ± 0.5  (4.8–7.1) | 19.6 ± 1.1  (18.2–22.0) | 5.6 ± 0.3  (5.1–6.0) | 19.5 ± 1.1  (16.9–20.9) | 6.4 ± 0.4  (5.8–7.0) | 20.0 ± 2.0  (17.2–23.7) |
| c’ | 7.8 ± 0.7  (6.0–8.9) | 1.7 ± 0.1  (1.4–1.9) | 8.6 ± 0.7  (6.8–9.5) | 1.8 ± 0.1  (1.7–2.0) | 10.2 ± 0.8  (9.0–11.8) | 2.1 ± 0.2  (1.8–2.3) |
| V or T | 59.3 ± 1.3  (57.2–63.1) | 65.7 ± 4.7  (57.8–75.3) | 47.7 ± 1.0  (45.7–49.6) | 70.3 ± 4.1  (61.2–76.4) | 45.4 ± 1.9  (40.6–48.1) | 57.7 ± 3.7  (50.7–63.5) |
| Lip diam. | 9.0 ± 0.7  (8.3–10.4) | 8.6 ± 0.9  (6.9–10.4) | 5.2 ± 0.5  (4.9–6.3) | 4.8 ± 0.4  (4.2–5.6) | Not given | Not given |
| Stoma diam. at the middle part | 3.1 ± 0.3  (2.8–3.5) | 2.5 ± 0.3  (2.1–2.8) | 1.4 ± 0.1  (1.3–1.5) | 1.4 ± 0.1  (1.3–1.5) | Not given | Not given |
| Stoma depth | 21.8 ± 0.9  (20.1–23.6) | 18.6 ± 1.1  (16.7–20.8) | 16.8 ± 0.8  (15.3–18.0) | 15.9 ± 0.6  (15.3–17.4) | 15 ± 0.8  (13–16) | 13 ± 0.8  (12–15) |
| Stoma depth/diam. ratio | 7.2 ± 0.7  (6.0–8.3) | 7.6 ± 1.2  (6.5–10.0) | 12.1 ± 0.4  (11.0–13.0) | 11.4 ± 0.4  (11.0–12.5) | Not given | Not given |
| Anterior pharynx length | 75 ± 2.7  (69–79) | 73 ± 3.9  (64–80) | 76 ± 2.4  (70–80) | 72 ± 1.9  (69–77) | 68 ± 2.9  (63–73) | 62 ± 3.0  (53–68) |
| Posterior pharynx length | 66 ± 3.1  (61–71) | 66 ± 4.4  (54–72) | 66 ± 4.3  (59–77) | 59 ± 2.5  (54–63) | 65 ± 2.7  (60–69) | 57 ± 2.9  (48–62) |
| Anterior/posterior pharynx length ratio | 1.13 ± 0.05  (1.03–1.25) | 1.11 ± 0.04  (1.02–1.19) | 1.16 ± 0.06 (1.01–1.29) | 1.22 ± 0.05  (1.13–1.30) | Not given | Not given |
| Median bulb (metacorpus) diam. | 18.3 ± 1.3  (16.0–20.1) | 17.8 ± 1.7  (13.9–20.1) | 18.1 ± 0.8  (16.7–19.4) | 15.9 ± 0.6  (15.3–16.7) | Not given | Not given |
| Basal bulb diam. | 15.4 ± 1.0  (13.9–17.4) | 16.4 ± 2.2  (11.8–19.4) | 16.2 ± 1.4  (13.9–18.8) | 13.2 ± 0.6  (12.5–14.6) | Not given | Not given |
| Nerve ring from anterior end | 94 ± 3.8  (86–101) | 93 ± 5.0  (82–101) | 97 ± 2.7  (90–101) | 95 ± 3.4  (88–104) | Not given | Not given |
| Nerve ring from posterior end of anterior pharynx | 14.1 ± 2.2  (11.4–19.4) | 16.5 ± 2.6  (12.6–22.9) | 17.0 ± 1.6  (13.7–20.6) | 19.7 ± 2.4  (14.9–24.0) | Not given | Not given |
| Hemizonid from anterior end | 119 ± 4.1  (110–125) | 120 ± 6.6  (109–133) | 121 ± 4.7  (114–129) | 119 ± 5.4  (109–129) | Not given | Not given |
| Hemizonid from posterior end of anterior pharynx | 39 ± 3.1  (33–43) | 43 ± 4.9  (35–54) | 41 ± 4.0  (34–48) | 44 ± 4.7  (35–53) | Not given | Not given |
| Excretory pore from anterior end | 125 ± 4.3  (115–135) | 127 ± 7.1  (112–141) | 128 ± 5.9  (119–141) | 128 ± 5.4  (118–141) | Not given | Not given |
| Excretory pore from posterior end of anterior pharynx | 46 ± 3.3  (40–53) | 51 ± 4.5  (43–61) | 49 ± 5.5  (35–61) | 52 ± 4.9  (43–61) | Not given | Not given |
| Maximum body diam. | 36 ± 5.5  (26–42) | 29 ± 4.3  (21–35) | 37 ± 5.2  (30–49) | 29 ± 2.0  (26–33) | 38 ± 3.9  (31–45) | 28 ± 2.5  (22–32) |
| Anterior ovary length | 181 ± 42  (118–243) | – | 161 ± 22  (132–217) | – | 186 ± 33  (146–273) | – |
| Posterior ovary length | 187 ± 33  (130–243) | – | 161 ± 31  (104–213) | – | 199 ± 35  (123–246) | – |
| Vulval body diam. | 35 ± 5.4  (26–42) | – | 36 ± 4.3  (30–45) | – | Not given | – |
| Testis length including *vas deferens* and reflexed part | – | 471 ± 51  (388–597) | – | 524 ± 45  (418–593) | – | 463 ± 46  (392–543) |
| Testis reflextion length | – | 47 ± 7.4  (31–62) | – | 56 ± 10.2  (45–95) | – | 39 ± 4.2  (31–46) |
| *Vas deferens* length | – | 202 ± 25  (146–243) | – | 240 ± 29  (177–287) | – | Not given |
| *Vas deferens* length occupying whole testis in % | – | 43.0 ± 4.3  (36.3–54.7) | – | 45.7 ± 3.8  (40.7–53.1) | – | Not given |
| Anal or cloacal body diam. | 17.9 ± 1.8  (14.6–20.8) | 21.0 ± 1.7  (18.1–27.1) | 19.9 ± 2.2  (17.4–26.4) | 21.3 ± 1.1  (18.8–23.6) | 20 ± 1.6  (17–23) | 19 ± 1.1  (17–22) |
| Tail length | 140 ± 13  (113–161) | 37 ± 1.9  (31–39) | 171 ± 13  (142–191) | 38 ± 1.7  (36–42) | 202 ± 19  (158–231) | 40 ± 3.0  (35–46) |
| Anus-phasmid distance in female | 18.2 ± 2.2  (15.3–22.9) | – | 18.8 ± 2.0  (15.3–22.9) | – | Not given | – |
| Spicule length (chord from manubrium tip to distal tip) | – | 36 ± 1.9  (31–40) | – | 35 ± 1.9  (31–38) | – | Not given |
| Spicule length (curved along the calomus-lamina complex) | – | 42 ± 1.6  (38–45) | – | 44 ± 1.9  (38–45) | – | 46 ± 1.9  (41–48) |
| Gubernaculum length | – | 18.4 ± 1.1  (16.0–20.1) | – | 20.8 ± 1.5  (18.1–23.6) | – | 23 ± 1.7  (19–27) |

All measurements are in μm and in the form: mean ± s.d. (range).
